# Sensitivity of plankton indices to lake trophic conditions

**DOI:** 10.1007/s10661-016-5634-3

**Published:** 2016-10-17

**Authors:** A. Ochocka, A. Pasztaleniec

**Affiliations:** Department of Freshwater Assessment Methods and Monitoring, Institute of Environmental Protection-National Research Institute, Kolektorska 4, 01-692 Warsaw, Poland

**Keywords:** Phytoplankton, Rotifera, Crustacea, Plankton indices, Lakes, Trophic conditions

## Abstract

Herein, we report the response of indices based on phytoplankton and zooplankton and their combination to different nutrient concentrations in lakes. The study was carried out in ten lakes in northeastern Poland. Integrated samples were collected from the epilimnion during the summer of 2012–2013. Secchi disk visibility (SD), total phosphorus (TP), total nitrogen (TN), and chlorophyll *a* were used as proxies for eutrophication. We calculated 16 plankton indices: two phytoplankton indices, six crustacean indices, five rotiferan indices, two zooplankton diversity indices, and one combined phytoplankton and zooplankton index. Among them, nine indices with the strongest correlations with TP were selected: percentage share of Crustacean species indicative of high trophy in the indicative group’s numbers (IHT_CRU_), percentage share of Rotifera species indicative of high trophy in the indicative group’s numbers IHT_ROT_, Crustacean ratio of biomass to numbers B/N_CRU_, phytoplankton trophic index (TI_TP+TN_), Margalef’s index, percentage share of cyclopoid biomass in total biomass of Crustacea (CB), Rotifera numbers (N_ROT_), biomass of Cyclopoida (B_CY_), and ratio of the cyclopoid biomass to the biomass of Cladocera (CY/CL). The sensitivity of the normalized values of these indices to proxies of eutrophication was tested. IHT_CRU_, IHT_ROT_, and B/N_CRU_ were the most sensitive and gave the strongest responses at lower TP concentrations (<35 μg/L). The phytoplankton trophic index, TI_TP+TN_, together with the zooplankton-based Margalef’s index and CB were very sensitive in both low (<35 μg/L) and high (>60 μg/L) TP conditions. On the other hand, N_ROT_, B_CY_, and CY/CL were slightly sensitive at low TP concentrations while their reaction was notable at high TP concentrations. A similar pattern of response was observed for TN concentration and SD visibility.

## Introduction

Plankton is a key component of pelagic ecosystems, forming the basis for most trophic webs. A strong relationship exists between the two main components of plankton communities—phytoplankton and zooplankton. Phytoplankton, which are the main producers of organic matter in the pelagic zone (Kawecka and Eloranta 1994), are also an essential source of food for zooplankton—directly for herbivorous animals and indirectly for detritus feeders. Eutrophication has a considerable impact on both plankton components, causing many changes in their abundance and species composition and affecting the relationships between them. Changes in the plankton community structure in relation to physicochemical parameters may be a first sign of a deterioration in the water quality. The application of plankton indicators to lake water quality assessment has a long tradition (e.g., Järnefelt 1952; Spodniewska 1978; Willén 1979; Reynolds 1980, 1984; Rott 1984; Karabin 1985a; May and O’Hare 2005; Padisak et al. 2006; Čeirāns 2007; Kane et al. 2009). The Water Framework Directive (WFD, EC, 2000) introduced the requirement for the assessment and classification of lakes based on the communities of organisms inhabiting them (i.e., phytoplankton, macrophytes, phytobenthos, benthic macroinvertebrates, and fish). The main goal of the WFD is to achieve “good ecological status” in all water bodies. Ecological status is an expression of the quality of the structure and functioning of aquatic ecosystems and is considered by measuring the deviation from non-impacted reference conditions, characteristic for each type of water body. Zooplankton, widely recognized as an important indicator of lake eutrophication (Hakkari 1972; Gannon and Stemberger 1978; Karabin 1985a; Andronikova 1996; Čeirāns 2007; Haberman et al. 2007; Jeppesen et al. 2011; Ejsmont-Karabin 2012; Ejsmont-Karabin and Karabin 2013; Haberman and Haldna 2014; Jekatierynczuk-Rudczyk et al. 2014), was not ultimately included as a component of the WFD—compliant lake assessment system. Therefore, scientific papers concerning the applicability of zooplankton indices in the context of the WFD are scarce.

The composition of zooplankton is affected both by changes in primary production, indicative of eutrophication, and by changes in the structure and abundance of the planktivorous fish community (Lampert and Sommer 2001). The zooplankton community is thus strongly affected by both “bottom-up” and “top-down” forces, and the development of appropriate indices would enable the assessment of eutrophication pressure as well as changes in the fish community (Mills et al. 1987). It seems reasonable to take into account both plankton elements (phytoplankton and zooplankton) in the ecological status assessment of lakes. Summer plankton communities are considered to be most useful for assessing the quality of lake water (Järnefelt 1952; Spodniewska 1978; Karabin 1985a; Wojciechowska et al. 2002; Ejsmont-Karabin and Karabin 2013; Jekatierynczuk-Rudczyk et al. 2014). Due to the limited variability in the abiotic conditions in the summer period, plankton development is mainly affected by the trophic properties of lakes. During the summer, zooplankton communities reach their highest abundance and diversity. It is also well documented that, especially in the late summer (July–September in the temperate zone), the stability of the phytoplankton community is at its highest and the species richness reaches its maximum, reflecting rather well the physical and chemical conditions in the lake (e.g., Eloranta 1986; Padisak et al. 2006).

The aim of this paper was to test the sensitivity of phytoplankton and zooplankton indices to the main proxies of eutrophication pressure in order to evaluate the effects of eutrophication on the studied lakes and their usefulness as indicators of lake ecological status in the context of the WFD requirements. We demonstrated the response to eutrophication and the consistency of selected planktonic indices (see Table 1), calculated on the summer data from lakes characterized by different trophy. We applied the newly developed phytoplankton trophic index, TI_TP+TN_ (Pasztaleniec 2016); the Q index—based on the functional traits of phytoplankton (Padisak et al. 2006); six crustacean zooplankton indices developed by Ejsmont-Karabin and Karabin (2013); five rotiferan indices described by Ejsmont-Karabin (2012), which have been calibrated for the trophic conditions of lake assessment; one combined measure—the ratio of zooplankton biomass to phytoplankton biomass; and two diversity indices based on zooplankton abundance, developed by Shannon-Wiener and Margalef (Margalef 1958; Shannon and Weaver 1963). Two phytoplankton indices were treated as a baseline method of ecological status assessment compliant with the WFD.

**Table 1:** Overview of plankton indices responding to eutrophication pressure

Plankton community	Index acronym	Index description	References
Phytoplankton	Q index	Phytoplankton assemblages index	Padisak et al. (2006)
	TI _TP+TN_	Phytoplankton trophic index	Pasztaleniec (2016)
Crustacean zooplankton	N_CRU_	Numbers of Crustacea [ind./L]	Karabin (1985a), Ejsmont-Karabin and Karabin (2013)
	B_CY_	Biomass of Cyclopoida [mg w. wt./L]	
	CB	Percentage of cyclopoid biomass in total biomass of Crustacea [%]	
	CY/CL	Ratio of the cyclopoid biomass to the biomass of Cladocera	
	IHT_CRU_	Percentage of species indicative of high trophy in the indicative group’s numbers [%]	
	B/N _CRU_	Ratio of biomass to numbers	
Rotifera zooplankton	N_ROT_	Rotifera numbers [ind./L]	Ejsmont-Karabin (2012)
	B_ROT_	Total biomass [mg w. wt./L]	
	TECTA	Percentage of form tecta in the population of *Keratella cochlearis* [%]	
	B/N_ROT_	Ratio of biomass to numbers	
	IHT_ROT_	Percentage of species indicative of high trophy in the indicative group’s number [%]	
Zooplankton	d	Margalef’s diversity index	Margalef (1958)
	H′	Shannon-Wiener’s diversity index	Shannon and Weaver (1963)
Phyto- and zooplankton	zoo/phyto	Ratio of zooplankton to phytoplankton biomass	

## Materials and methods

### Study area

**Fig. 1:**
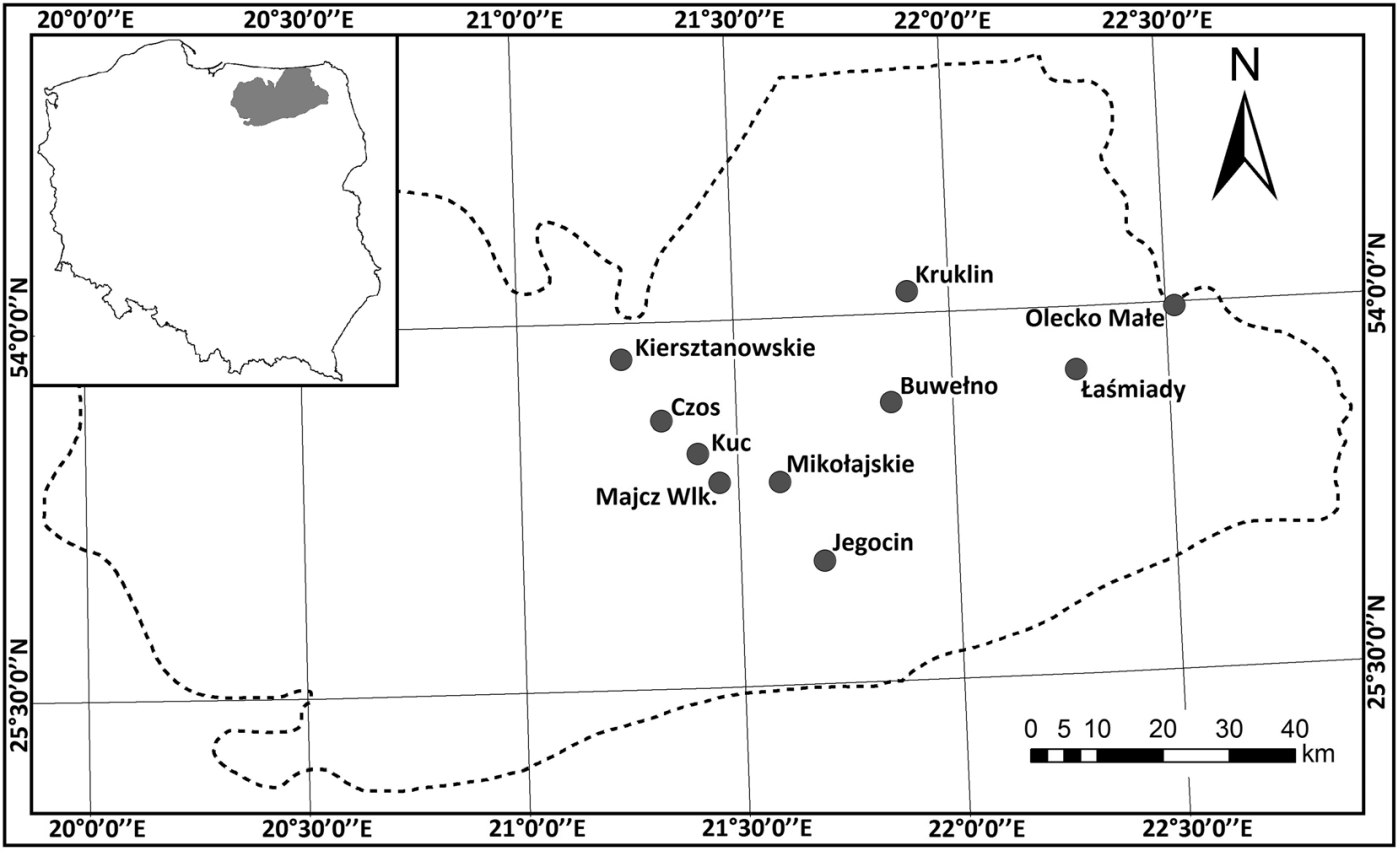
Localization of studied lakes in Masurian Lakeland

The studies were carried out in ten lakes situated within the Masurian Lake District in northeastern Poland within the limit of the last glaciation (Baltic) (Fig. 1). All of the lakes are lowland (<200 m a.s.l.) with a surface area exceeding 0.5 km^2^ and with highly colorless, alkaline water (>1.0 meq/L). They are stratified water bodies with a mean depth of at least about 5 m and a maximum depth ranging from 16.4 to 49.1 m (Table 2).

**Table 2:** Morphometric, physicochemical, and biological parameters of studied lakes

	Year	Jegocin	Kuc	Czos	Majcz Wlk.	Łaśmiady	Buwełno	Kiersztanowskie	Mikołajskie	Kruklin	Olecko Małe
Area [km^2^]		1.27	0.99	2.79	1.64	8.82	3.60	1.47	4.98	3.56	2.21
Volume [thous m^3^]		11,439.7	7949.1	31,012	9862.8	84,607.8	44,988.8	18,112.8	55,739.7	17,688	22,737.2
Mean depth [m]		9.0	8.0	11.1	6.0	9.6	12.5	12.2	11.2	4.9	10.3
Max. depth [m]		36.1	28.0	42.6	16.4	43.7	49.1	32.5	25.9	25.1	38.3
Temp [°C]	2012	20.4	22.1	22.0	22.8	22.0	22.5	22.8	19.1	22.5	22.0
	2013	20.2	19.2	18.7	19.0	21.1	20.5	18.7	18.8	21.0	19.8
pH	2012	7.4	7.5	7.3	7.5	7.7	7.7	7.6	7.1	7.8	7.9
	2013	8.4	7.4	7.3	7.4	7.9	7.8	7.6	7.8	7.8	7.8
Conductivity [μS/ml]	2012	223	223	344	309	371	408	408	346	395	400
	2013	190	201	320	278	350	369	369	316	368	386
SD [m]	2012	6.7	4.3	2.1	2.8	2.5	2.3	1.2	1.5	1.0	1.0
	2013	4.5	3.3	1.8	2.6	2.2	2.3	1.3	0.9	1.0	1.0
Chl-*a* [μg/L]	2012	4.3	6.5	6.6	6.2	25.9	4.3	16.4	21.3	43	58
	2013	2.2	4	8.9	6.3	20.9	4.4	22.3	30.6	48.9	64.2
TP [mg/L]	2012	0.020	0.021	0.034	0.028	0.036	0.025	0.047	0.035	0.048	0.073
	2013	0.010	0.023	0.058	0.057	0.051	0.041	0.046	0.034	0.123	0.069
TN [mg/L]	2012	0.77	0.83	0.99	0.81	0.99	0.86	0.96	1	1.16	1.48
	2013	0.74	0.79	1.14	0.6	0.87	0.94	0.86	1.1	1.38	1.22

Lakes listed in increasing trophy order

The lakes are affected by different anthropogenic pressures. Based on Hościło and Tomaszewska (2014), more than 60 % of the catchments of Lakes Olecko Małe, Kruklin, Kiersztanowskie, Buwełno, and Czos is agricultural land. There is slightly less agricultural land in the catchments of Lakes Łaśmiady (58 %) and Mikołajskie (48 %), whereas the catchments of Lakes Jegocin and Majcz Wlk. are dominated by forests and seminatural areas (scrubs and/or herbaceous vegetation), to the extent of 90 and 73 %, respectively.

### Data collection

In 2012 and 2013, during the summer stagnation period (July–August), integrated water samples were collected from the epilimnion of each lake. Over the two consecutive years, 20 samples were taken for physicochemical and plankton analyses. Samples for chemical and plankton analyses were taken using a Limnos type sampler (2.6 l capacity) at the deepest part of each lake, at 1-m depth intervals from the surface to the bottom of the epilimnion, and then pooled together. At the same time, the Secchi disk visibility (SD) was measured and field measurements of water temperature, pH, conductivity, and oxygen concentration were carried out using a YSI 650 MDS multiparametric probe (Ohio, USA). The chemical analyses of total values of phosphorus (TP) and nitrogen (TN) were performed using standard methods (Hermanowicz et al. 1999). The measurements of chlorophyll *a* concentrations involved filtering water with Whatman GF/C filters on the day of sampling, followed by extraction in 90 % acetone (Goltherman 1969) and determination by the spectrophotometric method (Nusch 1980). All of the physicochemical and biological data are presented in Table 2.

Samples for zooplankton analysis were concentrated using a plankton net with a 30-μm mesh size. Phytoplankton and zooplankton samples were fixed with Lugol’s solution and preserved in 4 % formalin. Microscopic analyses of the phytoplankton abundance and biomass were carried out using the Utermöhl method (1958). For counting, samples were transferred to a settling chamber (2.5 or 5 ml capacities were used, depending on algal density), and at least 100 individuals of the most numerous algae were counted per sample. Biovolumes were determined according to Hillebrand et al. (1999). For taxonomic analysis, additional samples were obtained with a 20-μm plankton net, and the phytoplankton species composition was determined under a light microscope (Zeiss, Axio Imager A2) on living and formalin-glycerin mixture-fixed samples. The zooplankton species composition was determined and individuals were measured under a Nikon ECLIPSE E200 light microscope. The crustacean zooplankton biomass was estimated on the basis of the relationship between the body length and body weight for each species (Balushkina and Vinberg 1979). The standard wet weight of rotifers was determined from the individual body weights, in accordance with Ejsmont-Karabin (1998). The species *Asplanchna priodonta* and *Leptodora kindti* were excluded from the analysis because of their large size, which was many times greater than that of either Rotiferan or Crustacean species.

### Statistical data analysis

During the preparation of the database for analysis, one sample (Lake Kruklin 2013) was recognized as an outlier based on multiple regression with the residue analysis and was excluded from future analysis. Therefore, 19 samples were used in further analysis.

Spearman rank correlation coefficients were used to calculate the relationships between 16 plankton indices and selected proxies of eutrophication (TP, TN, and SD). The concentration of total phosphorus was used for the latter analysis as it is widely considered as a major proxy of eutrophication pressure (Lyche-Solheim et al. 2013). In order to compare the sensitivity of the selected indices to eutrophication (i.e., those most strongly correlated with TP), the values of the indices were normalized to obtain values between 0 and 1 by dividing the index absolute values with the maximum value observed for each index in the dataset. It was assumed that for each index, 0 means the best quality and 1 means the worst quality. TP and TN concentrations as well as Secchi disk visibility were adopted as variables that best described the trophic conditions. We tested the sensitivity of the nine selected indices to the abovementioned eutrophication parameters using scatter plots using the distance weighted least squares smoothing model. Based on chlorophyll *a* concentration, the studied lakes were classified into “good” and “worse than good” ecological status according to Soszka et al. (2008) and the statistical significance of the differences between the values of the plankton indices in these two groups of lakes was determined using the non-parametric Mann-Whitney U test. All of the statistical analyses were carried out using STATISTICA 10.0 PL software.

## Results

### Physicochemical characteristics and trophic variables

The physicochemical parameters are presented in Table 2. The temperature varied in a narrow range from 18.8 to 22.8 °C. Considering the trophic parameters, Jegocin was the least fertile, as its TP and TN did not exceed 20 μg/L and 0.8 mg/L, respectively. In this lake, the maximum value of chlorophyll *a* concentration was only 4.3 μg/L, which was reflected in its high water transparency (6.7 m) and low phytoplankton biomass (0.7 mg/L). Low values of the trophic parameters were also found in Lakes Kuc, Buwełno and Majcz Wlk. The highest fertility was found in two lakes, Olecko Małe and Kruklin (Table 2).

### Phytoplankton community

**Fig. 2:**
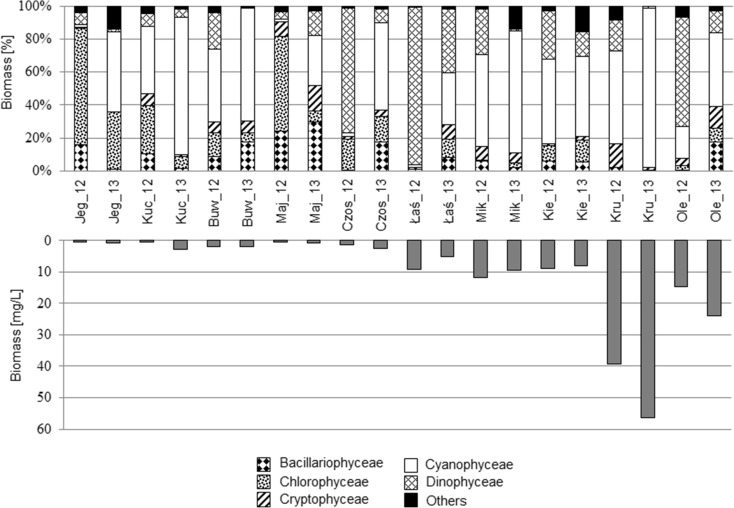
The taxonomic structure and biomass of phytoplankton (*Jeg* Jegocin, *Buw* Buwełno, *Maj* Majcz Wlk., *Łaś* Łaśmiady, *Mik* Mikołajskie, *Kie* Kiersztanowskie, *Kru* Kruklin, *Ole* Olecko Małe) investigated in 2012 and 2013

The lowest values of total summer phytoplankton biomass were noted in Lakes Jegocin, Kuc, Buwełno, Majcz Wlk., and Czos (ranging from 0.4 to 2.7 mg/L). Higher abundances were noted in Lakes Łaśmiady, Mikołajskie, and Kiersztanowskie (with a minimum value of 5.17 mg/L in Łaśmiady in 2013 and a maximum value of 11.86 in Mikołajskie in 2012) (Fig. 2). The largest phytoplankton biomass characterized Lakes Kruklin and Olecko Małe. In Lake Kruklin, the total biomass reached as much as 56.4 mg/L in the summer of 2013 against 39.1 in 2012. In Lake Olecko Małe, the total biomass was 14.7 and 24.1 mg/l, respectively, in 2012 and 2013. In total, 144 phytoplankton species were identified in the ten lakes, consisting of 86 genera, six main classes (Bacillariophyceae, Chlorophyceae, Cryptophyceae, Dinophyceae and Cyanophyceae), and 11 functional groups sensu Reynolds (1980, 1984)-C, F, H1, H2, J, K, Lo, P, S1, X3, and Y. A considerable percentage share of cyanobacteria occurred in all the lakes in at least one of the 2 years of the study (Fig. 2). In lakes with a lower total biomass, the dominant cyanobacterial species could be classified into functional group K (*Aphanothece*, *Aphanocapsa*), together with the large Dinophyceae-group Lo (*Ceratium*, *Peridinium*, *Aphanocapsa*, *Snowella*) or group H1 (*Anabaena*). In lakes with higher total biomass (about 10 mg/L or more), the functional groups Lo and H1 were often still dominant or subdominant, but filamentous cyanobacteria species (*Planktothrix*, *Limnothrix*, *Planktolyngbya*, *Pseudoanabaena*) belonging to the S1 group and *Gleotrichia* genera (H2 group) dominated the phytoplankton. Among green algae, colonial forms from the genera *Botryococcus*, *Oocystis*, and Coenoccoccus (functional group F) or single-celled organisms and coenobia of Chlorococcales, constituting group J (*Monoraphidium*, *Tetraedron*, *Pediastrum*, *Coelastrum*) or X3 (small unicells Chlorococales) were numerous.

Among Cryptophyceae and Dinophyceae clasess, Cryptomonas and Gymnodinium (Y group) were common in the most studied lakes.

### Zooplankton community

Altogether, zooplankton assemblages were composed of 52 species belonging to two main zooplankton taxonomic groups, Rotifera and Crustacea (including 13 species of Cladocera, five species of Calanoida, and three species belonging to the Cyclopoida). Rotifera was the most species-rich group, with 31 species being identified. The greatest species richness was noted in Lakes Kuc (33) and Jegocin (30), whereas the lowest number of species was found in Lake Kiersztanowskie-18 species and Lake Olecko Małe-19 species.

#### Zooplankton Crustacea

**Fig. 3:**
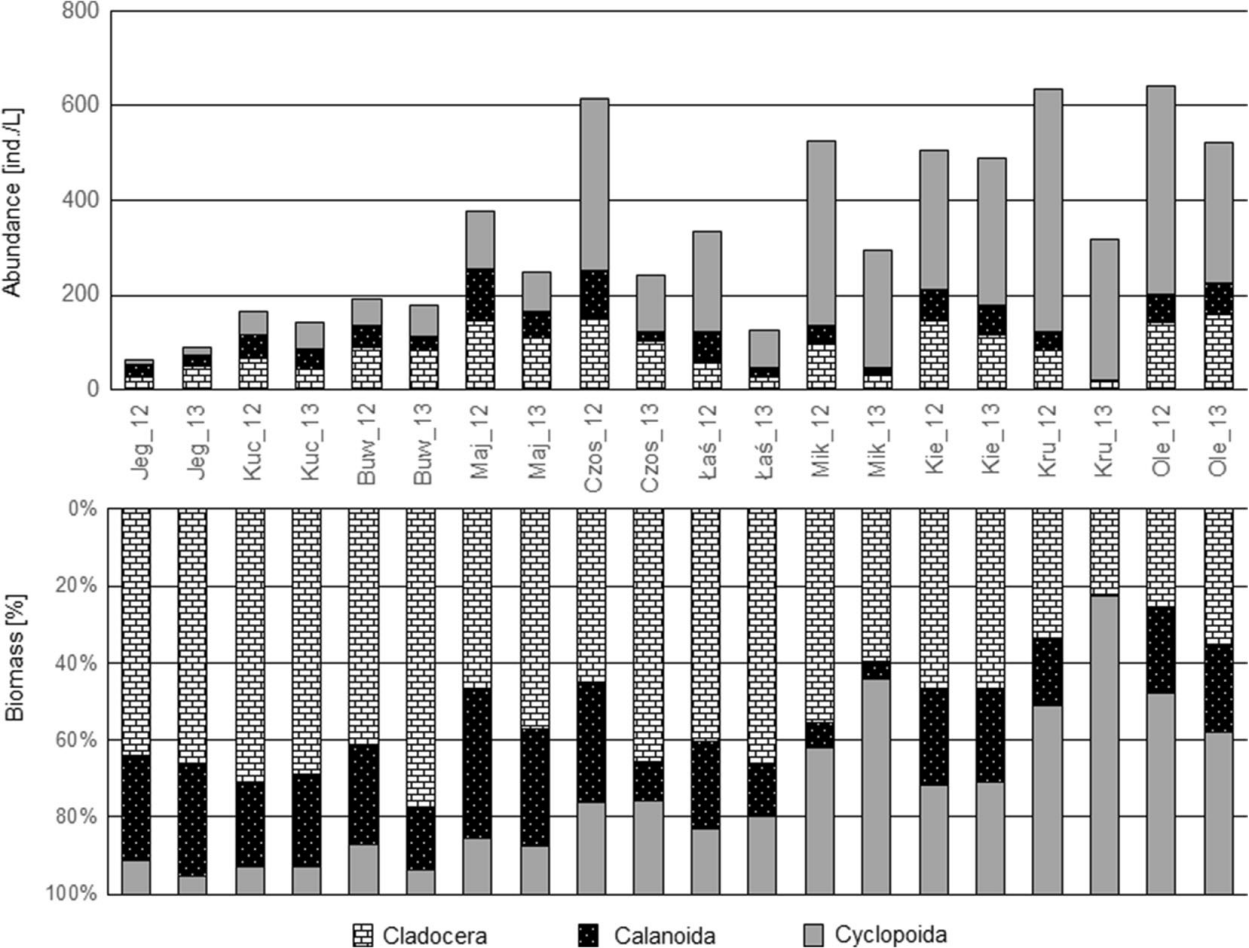
The abundance and biomass structure of Crustacean zooplankton in the studied lakes (Jeg, Buw, Maj, Łaś, Mik, Kie, Kru, Ole - refer in a key to the Fig. 2) investigated in 2012 and 2013

The highest abundance of Crustacea was observed in Lakes Olecko Małe, Kruklin, and Kiersztanowskie in both years (Fig. 3). The Cyclopoida group reached the largest densities in these lakes. The lowest density crustacean zooplankton (from 64 to 193 ind./L) was recorded in Lakes Jegocin, Kuc, and Buwełno (Fig. 3). In both years of investigation, Cladocera was the dominant group in these lakes, with its percentage share of the total crustacean biomass fluctuating from 62 to 78 %. A much lower share of Cladocera in the total crustacean biomass (from 22 to 40 %) was observed in Lakes Kruklin, Olecko Małe, and Mikołajskie (in 2013), which were dominated by Cyclopoida to the extent of 42 to 77 %*.*

#### Rotifera

**Fig. 4:**
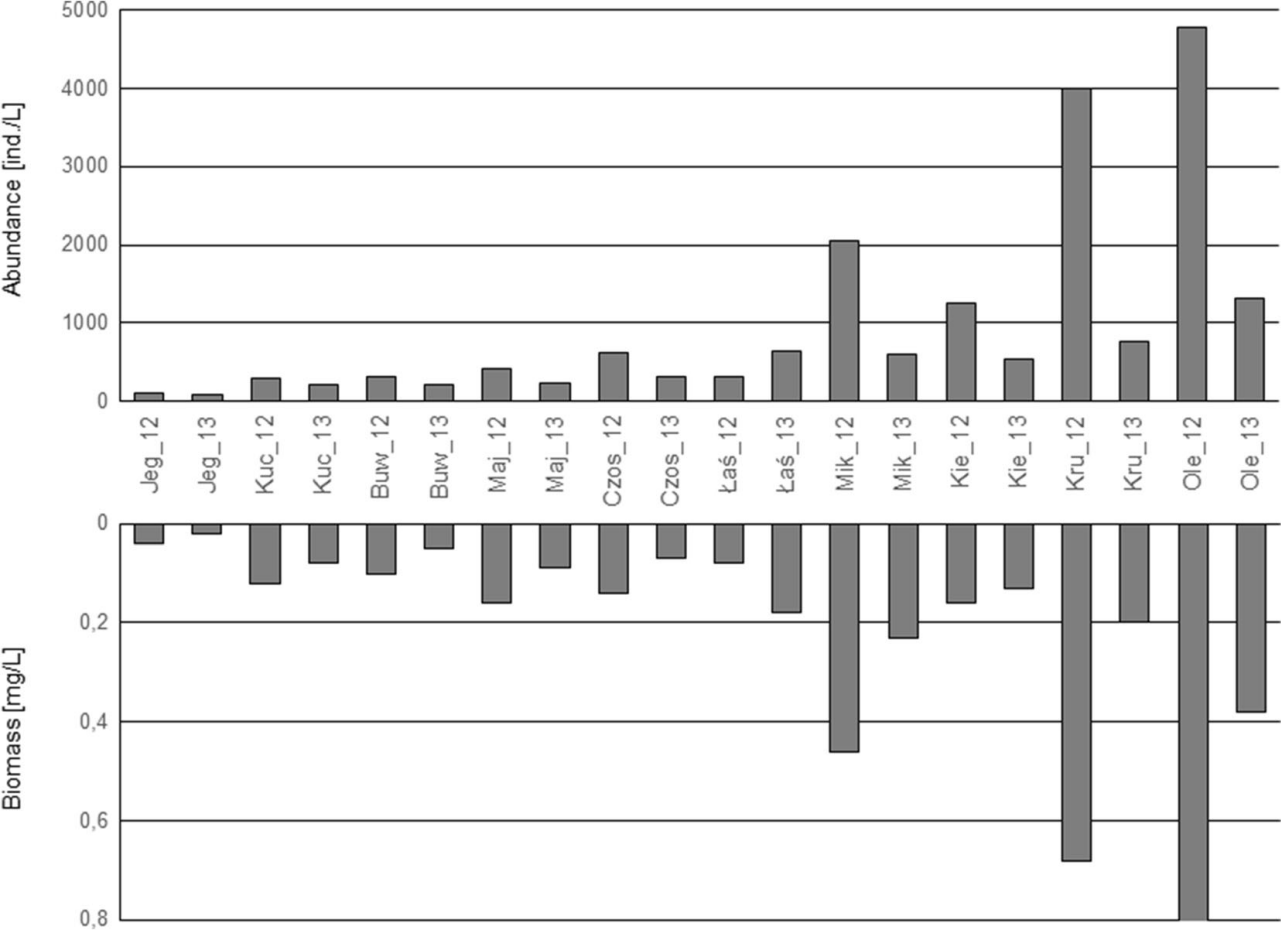
The abundance and biomass of Rotifera in the studied lakes (Jeg, Buw, Maj, Łaś, Mik, Kie, Kru, Ole - refer in a key to the Fig. 2) investigated in 2012 and 2013

The number of Rotifera ranged from the lowest in Lake Jegocin (114 ind./L in 2012 and 78 ind./L in 2013) to the highest in Lake Olecko Małe (almost 4800 ind./L and 1322 ind./L in 2012 and 2013, respectively). The lowest biomass values were recorded in Lakes Jegocin, Buwełno, Kuc, and Majcz Wlk. (from 0.02 to 0.16 mg/L), whereas the highest values were recorded in Lake Mikołajskie, Kruklin, and Olecko Małe (from 0.2 to 0.82 mg/L) (Fig. 4). Species characteristic of low trophy (*Ascomorpha ecaudis*, *A. ovalis*, and *Gastropus stylifer*) were noted in five lakes (Jegocin, Kuc, Buwełno, Majcz Wlk., and Łaśmiady). In the other lakes, these species were absent, with the exception of *A. ovalis* in Lake Czos (in 2012) and *A. ecaudis* in Lake Mikołajskie (in 2013), where their share of the abundance was small (0.5 and 0.3 %, respectively). Species with a considerable percentage share of the total abundance of Rotifera in these lakes were high trophy indicators. They included *Aneuropsiss fissa*, *Trichocerca pusilla*, and *Keratella cochlearis* f*. tecta*. Rotifera was the most abundant group with at least a 70 % contribution to the total zooplankton number in Lakes Olecko Małe, Kruklin, Łaśmiady, and Mikołajskie.

#### Phytoplankton indices

**Fig. 5:**
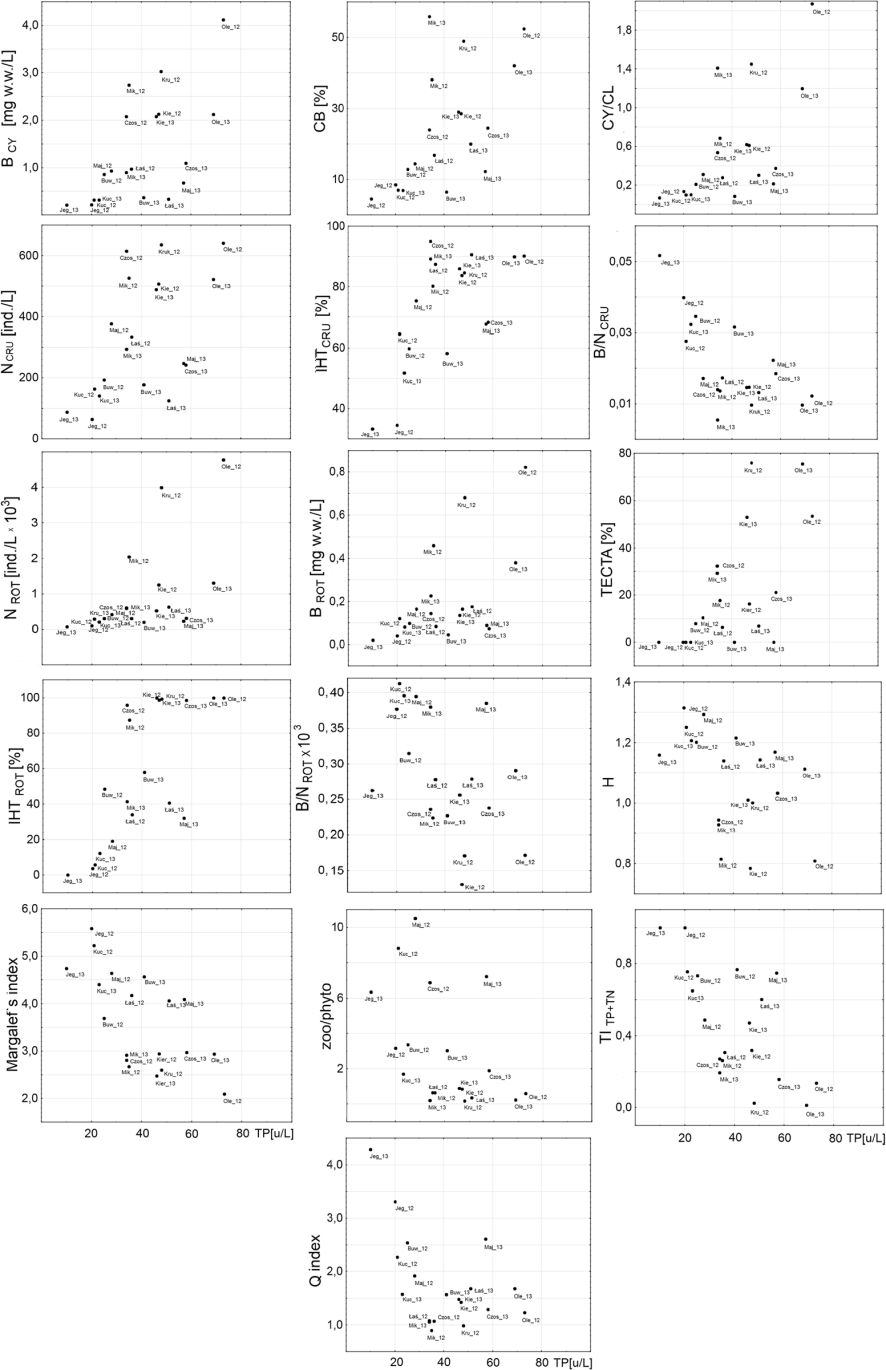
The relationship between TP concentration and Crustacean indices (N_CR_, B_CY_, CB, CY/CL, IHT_CRU_, B/N_CRU_), Rotifera indices (N_ROT_, B_ROT_, TECTA, IHT_ROT_, B/N_ROT_), zooplankton diversity indices (H′, Margalef’s index), phytoplankton indices (Q index, TI_TP+TN_), and combined plankton index (zoo/phyto)

Both of the tested phytoplankton indices, the Q index and TI_TP+TN_, reached their highest values (representing the best status) in Lake Jegocin, which was distinctly separate from the other indices in a plot of the values of the indices against increasing TP concentration (Fig. 5). The group of lakes characterized by the lowest values of phytoplankton indices generally consisted of Lakes Kruklin, Olecko Małe, and Mikołajskie. Nevertheless, the Q index did not vary greatly (the values of the Q index ranged between 0.8 and 1.4) in most of the lake-years in spite of the different TP concentrations noted. Higher values of the Q index (above 2.0) were achieved for Lakes Buwełno and Kuc in 2012 and Lake Majcz Wlk. in 2013. There was a more uniform distribution of the TI_TP+TN_ values among the studied lakes (Fig. 5).

#### Crustacean indices

The lowest values of the crustacean indices were noted for Lakes Jegocin, Kuc, and Buwełno, ranging as follows: N_CRU_—64–193 ind./L; B_CY_—0.21–0.86 mg/L; CB—4.5–12.8 %; CY/CL—0.07–0.21; IHT_CRU_—33–64 %, whereas the values of the B/N_CRU_ index ranged from 0.03 to 0.05. The highest values were noted in Lakes Olecko Małe, Kruklin, and Mikołajskie. The N_CRU_ index ranged from 294 to 641 ind./L, whereas the other indices varied as follows: B_CY_—0.9–4.1 mg/L, CB—38–56 %, CY/CL—0.7–2.1, IHT_CRU_—80–95 %. These lakes were also characterized by low values of the biomass to number ratio: B/N_CRU_—0.006–0.013. (Fig. 5).

#### Rotiferan indices

The lowest values of the rotiferan indices were noted in Lakes Jegocin, Kuc, and Buwełno in both study years. The N_ROT_ index ranged from 78 to 290 ind./L, the B_ROT_ index was below 0.09 mg/L, and the TECTA index was 0 % because *K. cochlearis tecta*, which is a typical form in eutrophic lakes, was absent. The IHT_ROT_ index ranged between 4 and 32 %. The values of the B/N_ROT_ ranged from 0.00038 to 0.00041. Lakes Olecko Małe and Kruklin were characterized by the highest values of all the tested rotiferan indices. The N_ROT_ index for Lake Olecko Małe reached a value of 1304 ind./L, whereas for Kruklin, it was almost 4800 ind./L. The B_ROT_ index ranged between 0.38 and 0.82 mg and the TECTA index ranged from 54 to 76 %, whereas the IHT_ROT_ index was 100 % in both cases. The values of the B/N_ROT_ index did not exceed 0.00029. (Fig. 5). The highest zooplankton diversity was observed in Lakes Jegocin, Kuc, Buwełno, and Majcz Wlk., whereas the Shannon-Wiener index reached values from 1.1 to 1.3 and Margalef’s index ranged from 3.7 to 5.6. The lowest values of these indices were recorded in Lake Olecko Małe in 2012 (the Shannon-Wiener index being 0.8 and Margalef’s index 2.1). The combined zoo/phyto index reached its highest values (an index value above 6) in Lake Majcz Wlk. in both of the studied years and in Lake Jegocin in 2013, whereas for the other lake-years, much lower values (the index being below 4) were found, while a zoo/phyto ratio close to zero was calculated for Lake Kruklin (in 2012) and Lakes Olecko Małe and Mikołajskie (in 2013).

**Fig. 6:**
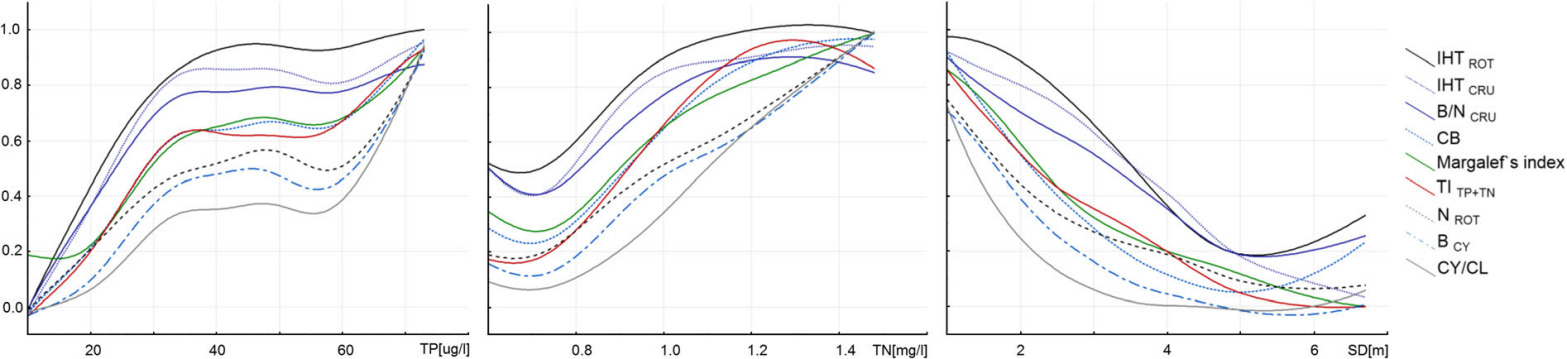
The *lines* represents the distance weighted least squares smoothing fitted model of relationships between normalized plankton indices and proxies of eutrophication (TP, TN, SD)

Among all of the tested indices, we selected nine that were the most strongly correlated with TP, TN, and SD (Spearman rank correlation coefficient ≥0.59, ≥0.68, ≥0.72, and at least *p* < 0.01, respectively) (Table 3). The relationships between the normalized tested indices and selected proxies of eutrophication (TP, TN, and SD) are shown in Fig. 6. The distance weighted least squares smoothing fitted model regression lines showed non-linearity for all the relationships in the analyzed spectrum of trophy. The response of all the indices to the eutrophication pressure had an approximately sigmoidal shape. There was a conspicuous sigmoidal response of almost all of the plankton indices to increasing total phosphorus concentration. The “S” shape in the SD model was the least obvious.

**Table 3:** The relationship between proxies of eutrophication (*TP* total phosphorus, *TN* total nitrogen, *SD* Secchi disk visibility) and all the tested indices (in order of statistical significance against TP)

	TP	TN	SD
IHT_ROT_	0.75***	0.78***	−0.87***
TI_TP+TN_	−0.66**	−0.88***	0.84***
B_CY_	0.65**	0.79***	−0.82***
CY/CL	0.62**	0.79***	−0.90***
B/N_CRU_	−0.62**	−0.77***	0.87***
Margalef’s index	−0.62**	−0.73***	0.89***
N_ROT_	0.62**	0.78***	−0.84***
CB	0.61**	0.82***	−0.92***
IHT_CRU_	0.59**	0.68**	−0.72***
TECTA	0.57*	0.81***	−0.88***
N_CRU_	0.54*	0.71***	−0.75***
H	−0.53*	−0.71***	0.84***
zoo/phyto	−0,52*	−0.72***	0.75***
B_ROT_	0.50*	0.66**	−0.77***
B/N_ROT_	−0.47*	−0.59***	0.57*
Q index	ns	−0.77***	0.71***

Spearman’s rank correlation coefficients R, marked with asterisks: ***p < 0.001; **p < 0.01; * *p* < 0.05

*ns* not significant

In the case of the TP model, below 10 μg of phosphorous, most indices (except for Margalef’s index) were close to 0. Their systematic growth was observed in the range from 10 μg TP/L to about 30–35 μgTP/L; at this concentration, the regression lines for all of the indices bent. In the TP range from 35 to 60 μg/L, the curves of all the tested indices flattened out but at very diverse levels, in the range from 0.3 to 0.9. Another inflection of the curve and a surge in the values of most indices took place at phosphorus concentrations above 60 μg/L. In general, three types of response of the tested indices to TP can be observed. The regression curves of three zooplankton indices, i.e., IHT_ROT_, IHT_CRU_, and B/N_CRU_, already reached high values (0.7–0.9) at low TP concentrations (below 35 μg/L), whereas at more than 35 μg/L TP, their values did not change greatly, falling within the range from about 0.8 to 1.0 (Fig. 6). The second group consisted of the phytoplankton TI_TP+TN_ index and two zooplankton indices: CB and Margalef’s index, which showed similar responses to growing trophy, already exceeding a normalized value of 0.6 at a TP concentration of 35 μg/L. The third group consisted of indices which were the least sensitive to increasing TP, i.e., N_ROT_, B_CY_, and CY/CL. In the TP range from 10 to 60 μg/L, their regression curves did not exceed a value of 0.6. Their values increased rapidly at total phosphorus concentrations close to the threshold of 60 μg/L. However, the response curves of all the three indices were clearly at lower levels than the others throughout the trophy spectrum.

In contrast to the TP model, at low total nitrogen (TN) concentrations, a large differentiation among the values of the tested indices could be seen, whereas at TN concentrations up to about 0.7 mg/L, the curves for most of the tested indices varied only slightly. The intensities of the response indices expressed by the normalized scale did not exceed 0.2 in the case of the indices CY/CL, B_CY_, N_ROT_, and TI_TP+TN_, varied around 0.3 for CB and Margalef’s index, and exceeded 0.4 for IHT_ROT_, IHT_CRU_, and B/N_CRU_. It was only over a TN concentration of 0.7 mg/L that the values of all of the indices grew and the further course of the curves of most of the tested indices was close to a linear response, with the exception of B/N_CRU_ and TI_TP+TN_, whose values did not show a tendency to increase at TN concentrations above 1.1–1.2 mg/L.

In the case of the dependence of the variability of the plankton indices on the water transparency gradient, at high SD values (of more than 5 m), the model curves of all of the tested indices showed no significant changes and the curves increased only when transparency reached lower values, such as 5–4.5 m (Fig.6).

The mean values of the plankton indices between the lakes classified as good and those with worse than good ecological status were significantly different (with the statistical significance levels ranging from *p* = 0.013 to *p* = 0.001) (Table 4).

**Table 4:** Nine selected plankton indices (in order of statistical significance against TP) and two classes of ecological status calculated based on the average spring and summer chlorophyll *a* concentration (Soszka et al. 2008)

Index	*N* = 10		*N* = 9		*p* value
	Good status		Worse than good status		
	mean	S.D.	mean	S.D.	
IHT_ROT_ [%]	37	37	78	30	0.013
TI_TP+TN_	0.66	0.28	0.26	0.19	0.006
B_CY_ [mg/L]	0.71	0.57	2.05	1.18	0.006
CY/CL	0.21	0.15	0.96	0.61	0.002
B/N_CRU_	0.029	0.012	0.012	0.004	0.001
N_ROT_ [ind./L]	276.1	154.4	1716.1	1616.7	0.002
Margalef’s index	4.3	0.9	3	0.7	0.005
CB [%]	12	7	37	14	0.001
IHT_CRU_ [%]	60	18	87	4	0.004

The ranges of the indices were also shown

## Discussion

Based on physicochemical analysis, it was found that the studied lakes represented a varied spectrum of trophic conditions, from the lowest trophic level of Lakes Jegocin, Kuc, Buwełno, and Majcz Wlk. to the most fertile lakes, such as Lakes Olecko Małe and Kruklin. The range of the values of the trophic indicators (Table 2) found in the analyzed lakes well reflects the trophic gradient typical for Polish lakes (Zdanowski 1983; Siuda et al. 2013; Jekatierynczuk-Rudczyk et al. 2014). The trophic conditions of the studied lakes were reflected by the quantity and quality of the plankton assemblages.

The study demonstrated a differentiated response of plankton indices to increased eutrophication as measured by higher TP and TN concentrations and lower Secchi disk visibility. The lowest normalized values of the indices were noted in the least eutrophic lakes, whereas the highest values of the indices were characteristic of the most fertile water bodies.

Zooplankton indices such as IHT_CRU_, IHT_ROT_, and B/N_CRU_ turned out to be the most sensitive (quickly reacting and reaching high values) to increased TP concentrations up to 35 μg/L, whereas they grew only slightly above this value. These indices responded similarly to increasing TN concentration, reaching high values already at low TN concentrations (from 0.8 to 1.0 mg/L), and their response broke down with a further deterioration in trophy. At the same time, the strength of the response of the abovementioned indices to increasing visibility was the greatest among all of the tested indices over the whole range of SD visibility recorded. In lakes with the lowest trophy, where the TN did not exceed 0.8 mg/L and SD was higher than 5 m, these indices did not vary.

One of the effects of eutrophication is an increased share of species that prefer eutrophic waters, belonging to the Crustacea and Rotifera (Karabin 1985a; Ejsmont-Karabin 2012; Ejsmont-Karabin and Karabin 2013). One of the three indices mentioned above, IHT_CRU_, turned out to be the most sensitive index at low TP concentrations. Karabin (1985a) indicates that the Crustacea preferring high-trophy waters primarily include species with a small body size, belonging to the order Cladocera, i.e., *Diaphanosoma brachyurum*, *Eubosmina thersites*, *Chydorus sphaericus*, and *Bosmina longirostris*, as well as *Mesocyclops leucartii* and *Thermocyclops oithonoides* of the order Cyclopoida. The results of our studies show that in the group of the lowest trophy lakes, even a slight increase in phosphorus concentration (from 10 to 35 μg TP/L) causes a significantly enhanced share of the Crustacea species indicative of high trophy (33–95 %) as a percentage of the abundance of all the indicator species. In turn, in lakes where the phosphorus concentrations exceeded 35 μg TP/L, the share of such species varied to a lesser extent (from about 60 to 90 %). In the case of the Rotifera, the species indicative of eutrophic conditions include *K. cochlearis* f. *tecta*, *K. quadrata*, *Pompholyx sulcata*, *Filinia longiseta*, *Anuraeopsis fissa*, and *Trichocerca pusilla* and the species belonging to the genus *Brachionus* (Karabin 1985a), which mostly feed on detritus, bacteria, and also small blue-green algae. Just as in the case of the Crustacea, even a slight increase in trophy causes an enhanced share of such Rotifera species in the abundance of all the indicative species (IHT_ROT_) in the range from 0 to 96 %. In most of the reservoirs where the TP concentrations exceeded 35 μg TP/L, the share of these species hardly varied and amounted to more than 90 %.

The B/N_CRU_ index was the third most sensitive index to higher TP concentrations, although it reached lower values than the abovementioned indices (i.e., the strength of its response is weaker). At low TP concentrations, this index showed the greatest variability (in the range from 0 to 0.7) (Fig. 6). In hardly fertile lakes, the abundance rose quickly in the range from 64 to 377 ind./L and was accompanied by only slight changes in biomass (2.5–6.6 mg/L). In the case of lakes where the TP exceeded 35 μg/L, the crustacean zooplankton reached a higher abundance (from 640 ind./L) and biomass reaching 8.5 mg/l. In these lakes, Cladocera species, *D. cuccullata*, *D. brachyurum*, *E. thersites*, *C. sphaericus*, and both juvenile and adult individuals of the Cyclopoida, *Mesocyclops leucarti* and *Thermocyclops oithonoides*, had a significant share in the total Crustacea biomass (>10 %). Eutrophication causes an enhanced abundance of crustacean plankton and reduces its mean body weight (Karabin 1985a) due to the dominance of filamentous cyanobacteria, which are not available as food to most species of the Crustacea (Haney 1987; Lampert 1987; DeBernardi and Giussani 1990). Cladocera with a small body size most often feed on bacteria and detritus (Karabin 1985b). In turn, adult individuals of Cyclopoida are to a large extent predators and their juvenile stages mostly feed on bacteria and detritus (Gliwicz 1969). Therefore, it is exactly those taxa that have a dominant share in eutrophic lakes. The share of large *Daphnia* species is substantially limited (Gliwicz 1977). In addition, the results of the studies by DeBernardi and Giussani (1990) demonstrate that small zooplankton species are less mechanically influenced by the presence of filamentous colonies of cyanobacteria. The opposite situation occurs in low-trophy lakes where the crustacean assemblage is dominated by species with a larger body size. However, their abundance is distinctly lesser than in fertile lakes. In these lakes, individuals with a large body size, belonging to the orders Calanoida and Cladocera, have a significant share in the biomass of the Crustacea (Fig. 3).

The development of the size structure of the zooplankton assemblages is considerably affected not only by the type and quantity of available food but also by the pressure exerted by planktivorous fish (Brooks and Dodson 1965; Gliwicz and Prejs 1977; Mills and Schiavone 1982; Jeppesen et al. 1997). The enhanced pressure from planktivorous fish that selectively feed on individuals with a large body size contributes to shifting the size structure of the Crustacea in more fertile lakes toward the dominance of individuals with a small body size. Both top-down and bottom-up impacts significantly influence the development of the size structure of zooplankton. Therefore, it seems that the B/N_CRU_ index may not only turn out to be useful for assessing the trophy of lake water but could also provide information on the character of the ichthyofauna and the intensity of predation by planktivorous fish.

Two of the tested zooplankton indices (Margalef’s index and CB) and the phytoplankton index (TI_TP+TN_) demonstrated a similar course in their distance weighted least squares smoothing model curves in their responses to both phosphorus and nitrogen concentrations and to deterioration of water transparency, the exception being Margalef’s index along the TP gradient, which was the only one of all the tested indices to reach a value of 0.2 at a concentration of 10 μg/L but showed no response in the range from 10 to 20 μg/L (Fig. 6). Moreover, the variability curves of the abovementioned indices in relation to phosphorus were similar and had a sigmoidal shape along the tested TP gradient. The distance weighted least squares smoothing model demonstrated that the relationship between TI_TP+TN_, the Margalef index, and CB and TP was linear up to approximately 35 μg/L, leveling off at a TP concentration higher than 40 μg/L. A similar, sigmoidal course for the regression curves of phytoplankton indices in relation to TP concentrations has been described by other authors (Ptacnik et al. 2009; Phillips et al. 2013). Moreover, the model flattened out at higher TP concentrations (greater than 100 μg/L). However, the model of the dependence between the abovementioned indices and TN and SD came closest to a linear one along the entire gradients.

The Margalef’s index reached its highest absolute values (Fig. 5) in the least fertile lakes, but the species diversity of zooplankton was diminished as they become more eutrophic. These results confirm the conclusions in the study by Jeppesen et al. (2011) that the species diversity of zooplankton diminishes with growing TP concentration. As demonstrated by Thakur et al. (2013), a deterioration in the quality of lake water caused by progressive eutrophication contributes to the elimination of plankton species with a small tolerance range and, simultaneously, to the dominance of tolerant species. These authors also stated that plankton diversity indices were good indicators of the degree of eutrophication. Analyzing the response of this index on the basis of its normalized values (Fig. 6), we can see that it is sensitive at both low (20–35 μg/L) and high (>60 μg/L) TP concentrations and along the whole TN gradient above 0.8 mg/L, as well as at Secchi disk visibilities from 0 to 6 m. The course of the response of the CB index, another zooplankton index, was very close to that of Margalef’s index. Data in the literature (Karabin 1985a; Ejsmont-Karabin and Karabin 2013) indicate that as they become more eutrophic, the share of Cyclopoida in the biomass of Crustacea increases. In the studied low-trophy lakes, Cyclopoida represented from 4.5 to about 13 % of the crustacean zooplankton biomass, whereas in the most fertile reservoirs, their share varied between 40 and 56 %. Thus, the results of our study confirm the conclusions of the abovementioned papers. The Cyclopoida flourish in fertile reservoirs, largely as a result of their manner of feeding and the type of their food. Cyclopoida select the food that they eat and their filtration apparatus serves to capture selected algal cells or detritus (Bednarska 2006), in contrast to the Cladocera, which filter water by passing it through their filtration chambers. In eutrophic lakes, the Cyclopoida can be seen to prevail over the Cladocera. This can be confirmed by analyzing the response of the CY/CL, another of the indices discussed here.

The N_ROT_, B_CY_, and CY/CL indices are the least sensitive to changes in nutrient concentrations and to reduction in the Secchi disk visibility. Their values grew significantly only at TP concentrations in excess of 60 μg/L, TN concentrations in excess of 0.8 mg/L, and Secchi disk visibilities of less than 3 m. There is comprehensive literature noting that there is a strong positive correlation between trophy growth and increasing abundance of the Rotifera (Karabin 1985a; Matveeva 1991; Yoshida et al. 2003; May and O’Hare 2005; Ejsmont-Karabin 2012). Our studies indicate that in strongly eutrophic lakes, the Rotifera abundance reaches extremely high values, almost 5000 ind./L. We also show that in these lakes, Cyclopoida biomass reaches high values—4 mg/L, representing more than 50 % of the whole crustacean zooplankton biomass. This confirms the results of Karabin (1985a) and Ejsmont-Karabin and Karabin (2013), showing that eutrophication causes an increase in the total biomass of Cyclopoida. At the same time, as trophy grows, the Cladocera biomass can be seen to diminish, since species with a small body size dominate in the assemblage. In his studies on the Masurian Lakes, Karabin (1985a) noted that eutrophication significantly contributed to decreasing dominance of the Cladocera in the zooplankton assemblage, while at the same time, the dominance of the Cyclopoida species increased. The response of the CY/CL index calculated from our data confirmed the conclusions from the earlier studies.

It can be concluded that the tested indices complement one another. Some indices (IHT_ROT_, IHT_CRU_, and B/N_CRU_) are more sensitive to the smallest trophy changes that unfold in relatively hardly fertile lakes and demonstrate the strongest response there. Therefore, they are suitable for the assessment of the water quality in low-trophy lakes. In turn, other indices (N_ROT_, B_CY_, and CY/CL) demonstrate the highest sensitivity to trophy changes that unfold in the most fertile lakes. These indices reach their highest values at the highest nutrient concentrations; thus, they are useful for the assessment of eutrophic lakes. The phytoplankton index, Margalef’s index, and CB show an intermediate response, since they are sensitive to a deterioration of the water quality in both low- and high-trophy lakes. Thus, it seems that the use of these indices to elaborate a multimetric index will enable a reliable and full assessment of lake water quality.

The composition and abundance of the Rotifera is mainly regulated by bottom-up forces, and they are less affected by fish predation, so the indices based on the Rotifera community structure give good information about the trophic state of a lake (Ejsmont-Karabin 2012). The community of Crustacea is strongly affected by both bottom-up and top-down forces; therefore, in addition to information on the trophic state of the lake water, the Crustacea indices can provide information on the character of the ichthyofauna (Mills and Schiavone 1982; Mills et al.^1987^).

It should be borne in mind, however, that some of the tested indices have certain limitations. The indices determining the share of species that prefer eutrophic lakes cannot be used to assess the lakes where these species are absent. In addition, it must be noted in particular that depending on their geographical/regional distribution, the species of the Rotifera and Crustacea that are indicative of high or low trophy can be different (Ejsmont-Karabin 2012; Ejsmont-Karabin and Karabin 2013). Therefore, in order to correctly use this index, each country should develop its own individual list of indicator species for low- and high-trophy lakes. Despite the limitations pointed out above, the comparison of mean values of the tested indices between two ecological status classes (“good status” and “worse than good status”) defined based on chlorophyll *a* indicates that the tested plankton indices corresponded with the ecological status assessment.

## Conclusions

Zooplankton indices have a strong indicator value and are equally sensitive to different trophic conditions as phytoplankton indices. Potentially, the most useful zooplankton indices include IHT_CRU_, IHT_ROT_, Margalef’s index, CB, B/N_CRU_, N_ROT_, B_CY_, and CY/CL. The elaboration of a plankton-based multimetric index using the indices considered here would make zooplankton an inexpensive indicator of the trophic state and ecological quality of lakes. Additionally, zooplankton are easily collected and may be readily identified. Our results confirm that zooplankton indices could be a promising tool for monitoring lake water quality.
